# Sex‐Specific Responses to Social Calls Shape Foraging Decisions in a Nectar‐Feeding Bat

**DOI:** 10.1111/nyas.70427

**Published:** 2026-10-21

**Authors:** Mirjam Knörnschild, Danilo Russo

**Affiliations:** ^1^ Museum Für Naturkunde, Leibniz Institute for Evolution and Biodiversity Science Berlin Germany; ^2^ Evolutionary Ethology, Institute for Biology Humboldt‐Universität Zu Berlin Berlin Germany; ^3^ Smithsonian Tropical Research Institute Panama City Panama; ^4^ Laboratory of Animal Ecology and Evolution (AnEcoEvo), Dipartimento di Agraria Università Degli Studi di Napoli Federico II Portici (Napoli) Italy

**Keywords:** Chiroptera, dominance, foraging choice, receiver‐dependent communication

## Abstract

Foraging in social animals involves balancing the benefits of social information against the costs of competition. In nectarivorous bats, social vocalizations are commonly produced at feeding sites, yet it remains unclear whether males and females respond differently to such signals. We investigated this by testing how alert calls linked to aggressive interactions and benign social calls influence foraging choices in bats (*Glossophaga mutica handleyi*). Bats changed their flower choices in response to playbacks, revealing marked sex differences. Males avoided flowers paired with alert calls, whereas females preferred them. Conversely, males showed no response to benign calls, whereas females were consistently drawn to flowers paired with social calls regardless of call type. Acoustic analyses revealed no differences between male and female alert calls, suggesting that these sex‐specific responses stem from receiver behavior rather than signal design. Furthermore, only dominant males approached flowers associated with alert calls, suggesting that competitive ability influences responses to conflict‐related signals. Overall, these results demonstrate that the same social signal can trigger different behavioral strategies in male and female bats. Our study underscores the importance of receiver‐dependent processes in shaping communication systems and shows that sex‐specific responses to social signals can develop without sex‐specific variation in signals.

## Graphical abstract

Playback experiments in a nectar‐feeding bat revealed contrasting responses to the same vocalizations, thus demonstrating that it depends on the receiver how social information is interpreted. Alert calls, often produced in aggressive contexts, showed no detectable acoustic differences between sexes, yet elicited different responses in males and females, highlighting that the same signal can influence different foraging strategies.

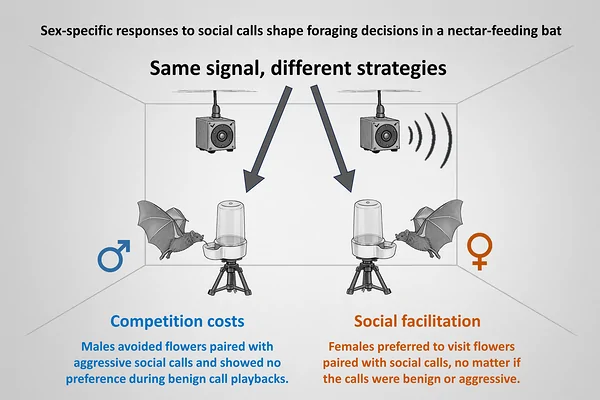

## 1 Introduction

Communication plays a crucial role in mediating social interactions among animals and shaping behaviors, such as mating, foraging, cooperation, and territoriality [1]. Social signals often serve multiple functions and can influence decision‐making in situations where the interests of signalers and different types of receivers do not fully align, creating potential conflicts over how signals should be interpreted and acted upon. Sexually selected signals, for instance, frequently attract females while repelling rival males [2]. This pattern is widespread: Typical examples include the songs and colorful plumage of male birds [3], the advertisement calls of orthopterans [4, 5] and anurans [4], and visual displays, such as push‐ups in some lizards [6], which function in mate attraction and male–male competition. Stag roaring in red deer is employed by females for mate choice [7] and by males in intrasexual aggressive encounters [8]. Similarly, in pipistrelle bats, social vocalizations that are part of the male advertising repertoire can also repel conspecifics from foraging areas, demonstrating how a single signal can serve both reproductive and competitive functions [9, 10, 11].

In contrast, signals primarily shaped by natural selection, such as contact calls [12] or alarm calls [13], are often expected to provoke similar responses across sexes because they are typically linked to shared survival or environmental pressures. However, this expectation does not always hold true. Even when males and females perceive the same signal, they may evaluate the associated costs and benefits differently, leading to sex‐specific behavioral responses [14, 15]. Such differences may stem from variations in reproductive strategies, ecological roles, physiological needs, and risk sensitivities between the sexes. For instance, males and females can respond differently to alarm calls, reflecting their distinct motivations and trade‐offs [16]. Therefore, understanding when social signals trigger sex‐specific responses is vital for interpreting the evolution of communication systems, especially in contexts where individuals must balance social information with the risk of competition.

Sex‐specific responses to the same signal have been documented across taxa, underscoring the receiver's role in shaping behavioral outcomes. For instance, in chaffinches (*Fringilla coelebs*), females prefer longer flourishes in male songs, whereas males respond more strongly to shorter flourishes, indicating sex‐dependent functions of specific song components [17, 18]. In sand fiddler crabs (*Uca pugilator*), males adjust claw‐waving behavior in the presence of females but not males, and only females respond to claw‐waving males [19, 20]. Together, these studies demonstrate that sex‐specific responses can occur whenever the fitness benefits of responding to a signal differ between males and females.

Competitive foraging contexts offer a particularly suitable framework for examining such divergence. In these contexts, individuals can use social cues or signals produced by conspecifics to locate resources, but these cues may also indicate the presence of competitors. In species that exploit ephemeral, patchily distributed resources, individuals must balance the potential benefits of social information against the risks of competition and aggression.

Nectar‐feeding bats are an excellent model for studying these trade‐offs. Each night, they visit hundreds of flowers, and nectar availability varies on short time scales, necessitating efficient decision‐making strategies [21, 22]. Their performance depends on spatial memory and learning, enabling individuals to track profitable feeding sites and optimize foraging routes [23, 24, 25, 26, 27]. At the same time, foraging decisions are shaped by social context, resource predictability, and competition, making nectar‐feeding bats a compelling system for investigating decision‐making under social constraints [21, 22].

The Pallas’ long‐tongued bat, *Glossophaga soricina*, is a small phyllostomid bat found in tropical habitats from northern Mexico to northern Argentina, where it roosts in tree hollows, caves, buildings, and bridges [28]. Recent research has shown that *G. soricina* is a species complex comprising at least four distinct species [29, 30]. Mainland Central American populations are currently referred to as *Glossophaga mutica handleyi* [30], but for simplicity we will refer to them as *G. mutica*. Pallas’ long‐tongued bats primarily feed on nectar and pollen but can switch to fruit and occasionally insects when floral resources are limited [31, 32, 33, 34]. Individuals often repeatedly visit the same feeding sites, suggesting trapline foraging [21, 31].

At low densities, both wild and captive *G. mutica* may temporarily monopolize concentrated food sources through aggressive aerial chases and vocalizations, indicating that acoustic signaling is an essential part of competitive interactions at feeding sites [31, 35, 36]. Anecdotal evidence suggests that captive male *G. mutica* defend food sources more aggressively against other males than against females and that male dominance status may influence nectar intake [37]. In *G. mutica*, alert call production tends to increase with agitation [38], and alert calls often accompany food‐related aggression and precede aerial chases that frequently result in displacement [25]. These observations suggest that alert calls may function as signals of heightened competitive activity at feeding sites, rather than simply being associated with ongoing interactions. As both sexes produce alert calls, it remains unclear whether these calls carry sex‐specific information and whether males and females respond differently to them during foraging.

To address these questions, we conducted playback experiments with captive *G. mutica* and complemented them with acoustic analyses and dominance assessments. We tested four hypotheses: First, if alert calls suggest a higher risk of conflict at a feeding site, bats will modify their flower choice in response to alert call playbacks. Second, if the perceived costs of confrontation depend on competitive ability, dominance rank will account for variation in response strength to alert call playbacks within each sex. Third, if alert calls serve as sex‐specific signals, we predict consistent structural differences between calls produced by males and females. Finally, if sex differences in behavioral responses are specifically linked to conflict‐related information rather than to social acoustic activity itself, responses to benign social calls will differ from those to alert calls and show reduced or altered sex‐specific patterns.

## 2 Materials and Methods

### 2.1 Study Subjects

We used two captive breeding colonies of *G. mutica* located in Germany, one for recording alert calls and benign calls (in 2008), and another for playback experiments and observations of dominance hierarchies (in 2010). The founding individuals of these colonies had been captured in Southern Mexico in 1988. Bats were housed in climate‐controlled rooms (26–28°C, 60%–75% relative humidity) with a reversed 12‐h photoperiod. They were fed ad libitum on honey water enriched with pollen or milk powder (Aletemil, Alete, Nestlé, Germany) and a nectar substitute (Nektar‐Plus, Nekton, Pforzheim, Germany). Bats were briefly captured using padded hand nets and transferred in soft bags between their housing and experimental rooms, where they remained for less than 60 min. For the playback and dominance hierarchy experiments, individuals were marked with removable flexible plastic collars with numbered rings (AC Hughes, England, size XCS) and small pieces of reflective tape attached to them. Husbandry was permitted by the respective state authorities, and the work was carried out in compliance with national legislation and university ethical standards.

### 2.2 Alert Call Measurements

We recorded alert calls from various all‐male and all‐female groups of *G. mutica* (10 different groups per sex and each with 4 individuals) housed temporarily in a 3.8 × 2.1 × 2.5 m^3^ flight room. Recordings were conducted from February to April 2008, with one recording night per group (20 nights in total). Both sexes produced two known alert call types year‐round (Type 1 corresponds to GS2 and Type 2 to GS4 as described in [35]; the species *G. soricina* in this article has been renamed into *G. mutica*); both types are monosyllabic. Calls were recorded using an Avisoft USG 116Hm system (sampling rate: 500 kHz, resolution: 16 bit, frequency response: 1–200 kHz ±3 dB) and a Toshiba Satellite 5200‐701 laptop running Avisoft‐RECORDER (v4.0, Avisoft Bioacoustics, Glienicke, Germany). Recordings of individual males and females were not conducted because the bats did not produce alert calls when alone, and, when kept in groups, it was not possible to identify the calling individual, as the bats vocalized in flight. Therefore, we selected one call per type per group for analysis to ensure representation across individuals, confirming that the alert calls originated from different individuals. Ten alert calls of each type were chosen for males and females.

Acoustic analyses were performed using Avisoft‐SASLab Pro (v5.2, Avisoft Bioacoustics, Glienicke, Germany). Sonograms were produced with a 1024‐point fast Fourier transform (frame size: 100%, Hamming window: 87.5% overlap, bandwidth: 635 Hz, frequency resolution: 488 Hz, time resolution: 0.256 ms). The start and end of calls were identified automatically (−12 dB relative to peak frequency). We measured 26 acoustic parameters, including four spectral parameters (peak frequency, minimum and maximum frequency, and bandwidth) at five evenly spaced locations across the call (resulting in 20 spectral parameters representing call curvature), and calculated their average over the entire call. Additionally, two temporal parameters were measured (duration and time to maximum amplitude). Principal component analysis (PCA) with varimax rotation was performed on the 20 spectral parameters describing call curvature, yielding four principal components (PCs) with eigenvalues >1, which explained 89.1% of the variance. The suitability of PCA was verified using Kaiser–Meyer–Olkin and Bartlett's tests. A MANOVA was conducted to assess sex‐related differences, with the four PCs and six other parameters as dependent variables, and sex and call type as fixed factors. We could not test for sex‐specific differences in benign calls (even though both sexes utter them) because their production could not be elicited as easily as alert calls, which were produced by placing same‐sexed bat groups in an unfamiliar room and subsequently entering this room.

### 2.3 Recording of Playback Stimuli and Generation of Playback Files

The playback experiment (Figure 1) was designed to mimic the vocal activity of several conspecifics (both alert and benign) at a feeding site. Alert and benign calls were recorded from a mixed‐sex group (*n* ≈ 100) over 12 nights across 2 months (September–October 2008), ensuring broad representation of individuals of both sexes. Bats were habituated to the presence of human observers, and behavioral observations during recordings were conducted with a nightscope (Litton Monocular M911). The behavioral context of alert calls was unmistakable: Whenever bats were mildly agitated, for example, because of a disturbance at their roosting place or conflict over food, they produced alert calls in flight. Their lower frequencies are in the audible range, which further facilitated observations. Benign calls were produced when bats were roosting side by side in a generally affiliative context but without any direct interactions.

**FIGURE 1:**
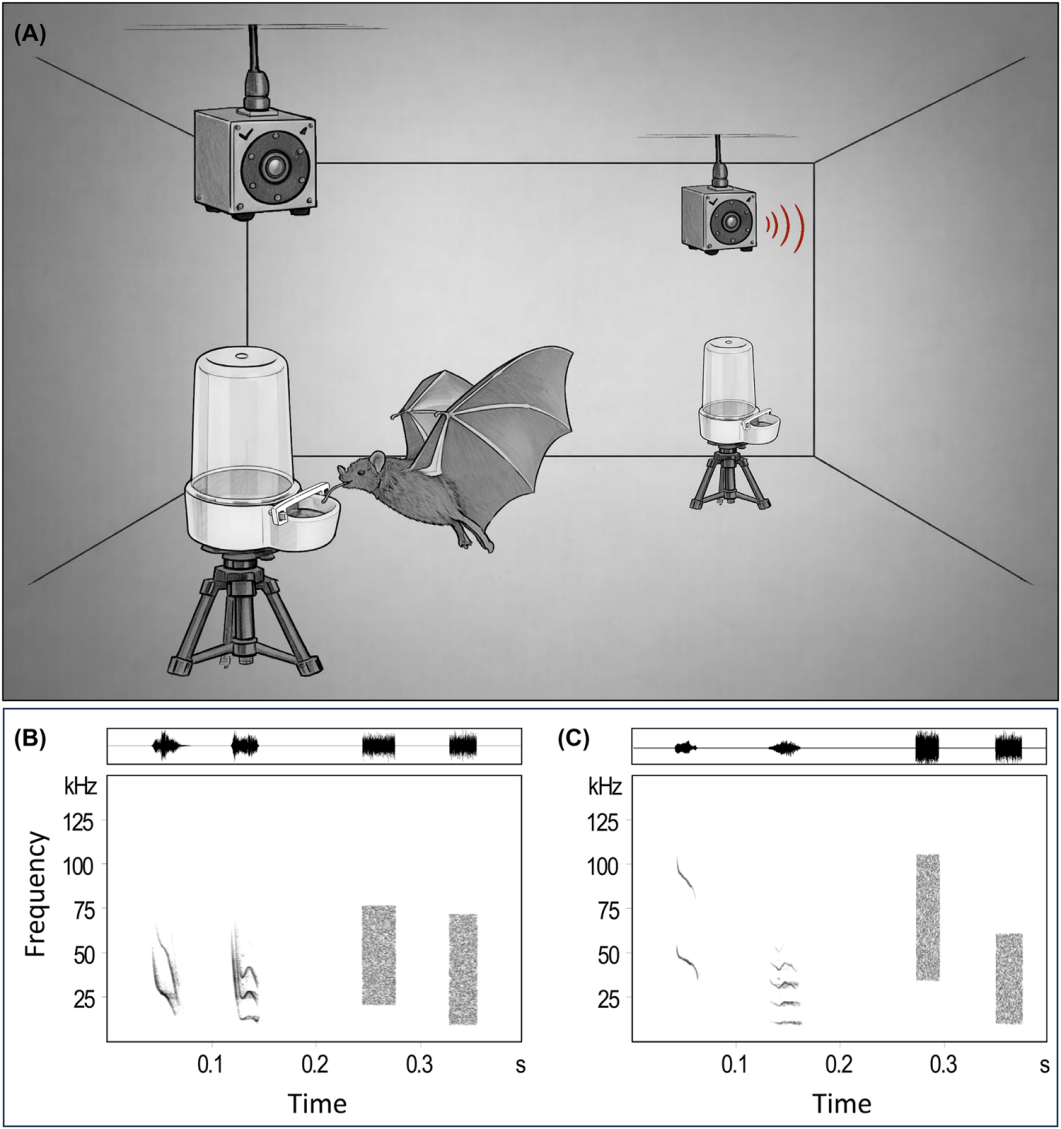
Experimental room with two feeders equipped with light barriers and two ceiling speakers in opposite corners (A). Spatial positions were reversed after each trial. Example playback stimuli used in the alert call (B) and benign call (C) experiments. Both oscillograms and spectrograms are shown. In these playback experiments, two vocalization types, alert calls (B) and benign calls (C), along with their respective noise controls, were broadcast. Spectrograms were generated using a 1024‐point fast Fourier transform, with a frame size of 100% and a Hamming window with 87.5% overlap.

As no sex differences were observed in alert calls, we used 87 high‐quality alert calls (Type 1: 41, Type 2: 46) and 93 benign calls (Type 1: 48, Type 2: 45; Type 1 corresponds to GS9 and Type 2 to GS15 *sensu* [35]) for playback. All calls used for playback stimuli were recorded exclusively in one colony, ensuring that focal bats in the second colony had no prior exposure to stimulus donors.

We carried out two playback experiments to examine whether alert or benign calls influenced the foraging behavior of individual male and female *G. mutica*. Each experiment compared vocalizations to silence and included white noise controls to ensure that the focal bats responded to the broadcast calls rather than to generic noise. Playback trials lasted 10 min each. One complete playback experiment comprised four consecutive playback trials. Each bat experienced only one playback experiment per night. Experiments always started 1 h after the onset of the bats’ dark cycle, to give them time to forage in their colony before the experiment.

Each unique playback file contained 100 stimuli (i.e., alert calls, benign calls, or their respective white noise controls), each separated by 2–8 s of silence. Alert calls were longer than benign calls (alert call Type 1: 30.4 ± 1.1 ms; alert call Type 2: 26.0 ± 2.3 ms; benign call Type 1: 11.9 ± 3.3 ms; benign call Type 2: 24.0 ± 5.4 ms; [35]), but the variable silent intervals between stimuli ensured that the duration of the four treatments was equivalent (600 s each). A computer randomly selected 100 stimuli from the existing pool of calls, with some stimuli repeated in each playback file. We regarded this as a minor drawback given the large number of stimuli (100) per trial. The stimuli were created using Cool Edit 2000 (Syntrillium, Phoenix, AZ, USA), bandpass filtered (5–100 kHz), and normalized to 75%. White noise stimuli were generated to match the frequency and duration of vocalizations.

### 2.4 Design of Playback Experiments

We tested 16 bats (8 males, 8 females) across two playback experiments. Each focal bat was placed in a 2.4 × 4.8 × 2.2 m^3^ flight room that contained a ceiling roost and two artificial flowers positioned at opposite corners, 4.4 m away from each other (Figure 1). The flowers provided nectar ad libitum (Nectar‐Plus, Nekton, Pforzheim, Germany). One flower was paired with an ultrasonic speaker (Avisoft ScanSpeak; frequency range 1–120 kHz ±12 dB, amplified via Avisoft Ultrasonic Power Amplifier), whereas the other featured a dummy speaker that was silent but resembled the real speaker. All playback stimuli were broadcast at 70 dB peSPL at 10 cm. *Glossophaga* sp. has faint echolocation calls (∼92 dB peSPL re 20 µPa at 10 cm [33]), and their social calls appear to be also low amplitude even though measurements are lacking so far. Focal bats probably heard the calls broadcast at the playback flower when approaching the silent flower, albeit much softer because they were further away from the source. This mimics a natural foraging situation where, for example, some branches of a flowering tree are disputed over whereas others are not.

Each experiment involved four randomized playback trials: two with alert or benign calls versus silence, and two with white noise versus silence (Figure S1). An experiment started as soon as the bat was placed into the ceiling roost of the experimental flight room and the lights were turned off. In the first trial, the speaker played alert calls (or benign calls in Experiment 2), with the dummy speaker serving as the control (silence). After each trial, the flowers and speakers were moved to opposite corners to control for potential side preferences. The focal bat retreated to its ceiling roost during the repositioning and resumed foraging once the flight room was vacated and the lights were turned off again. After the second trial, white noise was broadcast by the speaker, whereas the dummy speaker remained silent. The same procedure was repeated after reversing the positions of the flowers and speakers to further control for side preferences. The order of stimulus presentation (alert call or benign call first; white noise first) was balanced across trials. Bats were immediately returned to their colony after testing. The experimental design included three levels: (1) stimulus presence versus silence, (2) noise versus silence, and (3) spatial bias (original position vs. spatially reversed position).

### 2.5 Response Variables

Flower visits were recorded using custom‐made infrared light barriers (infrared retroreflective photoelectric sensor SICK WL9‐P13, connected to a personal computer via the LPT port and logged with custom Turbo Pascal 5.0 software at 10 ms temporal resolution) positioned on each artificial flower. We counted an interaction as a flower visit only if it lasted longer than 50 ms, to exclude brief contacts or passing interactions. We recorded the number of flower visits and measured the latency to the first flower visit in each playback trial for each focal bat. When a bat did not visit any flower during a playback trial, we assigned the maximum possible latency (600 s). This system has been successfully used to track flower visits of Glossophagine bats in previous studies [39, 40, 41].

To analyze behavioral responses, we fitted separate mixed‐effects models for each sex, call type, and playback condition. The number of flower visits was analyzed with negative binomial mixed models, and latency to first flower visit with linear mixed models fitted to log‐transformed latency values (log(1 + *x*)). In all models, flower (playback vs. silent) and repetition (1 vs. 2) were included as fixed effects, and bat identity as a random intercept. Inference was based on model‐based pairwise contrasts between playback and silent flowers derived from estimated marginal means. For flower visits, we report playback/silent visit ratios, whereas for latency we report contrasts on the log‐transformed scale. Analyses were conducted in RStudio (2025.09.2) using the packages *glmmTMB*, *lme4*, *emmeans*, and *DHARMa*.

To assess individual consistency across repetitions, we calculated separate preference scores for flower visits and latency and quantified agreement between Repetitions 1 and 2 within each sex, call type, and playback condition using Spearman rank correlations and intraclass correlation coefficients. Full calculation details and repeatability results are provided in the ESM (Table S1). Repeatability across the two repeated trials was assessed using the *cor.test()* function from the base R package *stats* and intraclass correlation coefficients estimated with the *lme4* package.

### 2.6 Establishing Dominance Hierarchies

After completion of the playback experiments, we established dominance hierarchies separately for males and females by observing same‐sex bats in an experimental room (5.8 m × 2.8 m × 2.5 m) equipped with a roost and an artificial flower. Dominance interactions were recorded using three infrared cameras (Sony HDR‐SR12E, DCR‐SR35E, DCR‐HC17E) for up to 1 h per trial. One bat in each pair was temporarily marked with a small stripe of reflective tape on its neck collar to differentiate individuals. The choice of which bat to mark was made randomly for each encounter, ensuring no bias in the marking process and that no negative effects from the marking were observed. Dominance was determined on the basis of the outcomes of dyadic interactions. The bat exhibiting the most dominant behaviors (cumulative counts over the observation period) was deemed the winner. Dominant behaviors included in our counts were aerial chases, hovering in front of and/or attempting to land on a roosting conspecific, attempted forearm strikes, and biting attempts while hanging next to each other. Bats were monitored closely, and if one bat was assumed to display actual physical aggression (biting or forearm strikes), the trial was immediately terminated and not repeated with that dyad. Some dyads were observed multiple times (up to six times) over 4 months, whereas others were tested only once. Despite repeated testing, some bats never interacted, and these missing interactions were recorded as Na/N in our dataset. Exemplary video material of dyadic interactions is provided in the ESM (Videos S1–S3).

We calculated David's score (DS) values [42, 43] to quantify individual dominance relationships, as has been done by previous studies [44, 45, 46, 47]. Dyadic interactions were organized into matrices for males and females (Tables S2 and S3), where rows indicated the number of wins (instances of one bat being dominant over another) and columns indicated losses (instances of one bat being subordinate). Higher DS values denote greater dominance. DSs were calculated from the matrixes of observed winner–loser interactions using the *DS()* function of the R package *EloRating*. These scores (Table S4) were then used to assess whether individual variation in responses to playback stimuli was associated with competitive ability within each sex.

We examined whether individual variation in dominance status was associated with behavioral performance by fitting separate univariate linear regression models, with DS as the independent variable and either the cumulative number of flower visits or latency to the first flower visit as the dependent variable. To avoid conflating potentially different response types, regressions were calculated separately for each playback type and sex. Model fit was assessed using *R* ^2^ and *F* statistics. Regression models were fitted using the *lm()* function in the base R package *stats*.

## 3 Results

We observed significant acoustic differences between the two alert call types, but no differences between males and females (MANOVA with 10 acoustic parameters as dependent variables and call type and sex as fixed factors; call type: *F*_10,27_ = 72.196, *p* < 0.001, η^2^ = 0.964; sex: *F*_10,27_ = 0.499, *p* = 0.875, η^2^ = 0.156; call type × sex: *F*_10,27_ = 0.630, *p* = 0.775, η^2^ = 0.189; Table S5, Data S1). These findings indicate that sex‐specific differences in alert calls are very unlikely.

Behavioral responses to alert calls differed between male and female bats (Data S2). Both sexes exhibited clear differences in their responses to call playbacks. Males avoided flowers where alert calls were broadcast and preferred to feed from silent flowers (negative binomial mixed model; estimated playback/silent ratio = 0.17, *z* = −6.43, *p* < 0.001; Figure 2A), whereas females were attracted to alert calls and favored flowers with alert call broadcasts over silent ones (ratio = 6.57, *z* = 6.05, *p* < 0.001; Figure 2B). In the corresponding white noise control, neither sex showed a significant difference in flower visits (males: ratio = 0.60, *z* = −1.75, *p* = 0.080; females: ratio = 1.12, *z* = 0.75, *p* = 0.451). When benign social calls were broadcast, females again preferred playback flowers over silent ones (ratio = 2.58, *z* = 4.48, *p* < 0.001; Figure 2D), whereas males showed no preference (ratio = 0.73, *z* = −1.72, *p* = 0.085; Figure 2C). In the corresponding white noise control, neither sex differed in their response to playback and silent flowers (males: ratio = 1.16, *z* = 0.82, *p* = 0.414; females: ratio = 0.94, *z* = −0.22, *p* = 0.830). Repetition did not significantly affect flower visits in any model (all *ps* ≥ 0.366).

**FIGURE 2:**
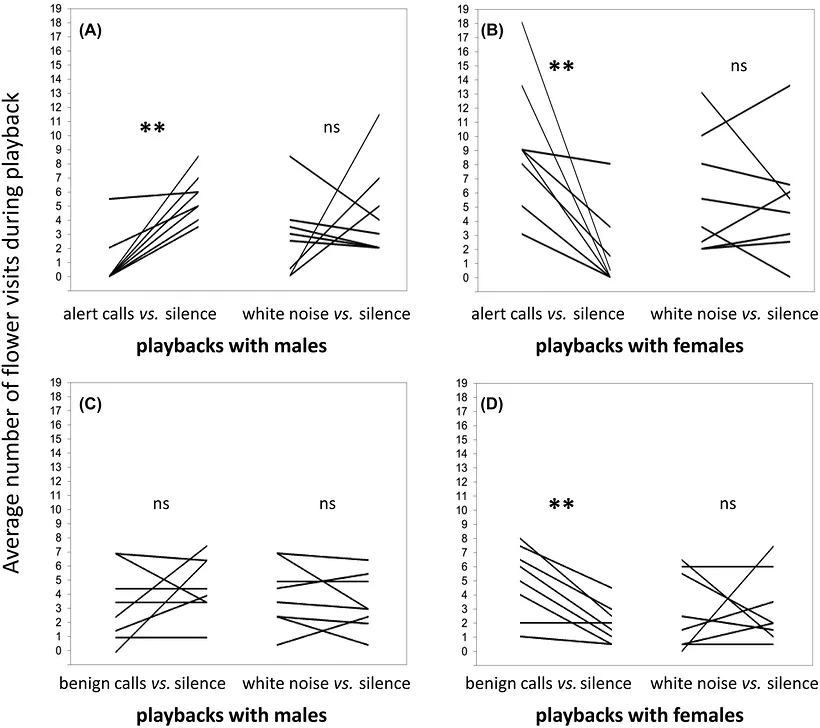
Average number of flower visits by focal bats during two playback experiments. In the alert call experiment, males avoided foraging at flowers where alert calls were broadcast (A), whereas females preferred foraging at these flowers (B). In the benign call experiment, females again preferred foraging at flowers where benign calls were broadcast (D), whereas the playback did not affect males' foraging behavior (C).

Latency to the first flower visit showed the same sex‐specific pattern (Figure S2). Males visited silent flowers more quickly than those where alert calls were broadcast, if they visited the latter at all (linear mixed model; contrast estimate = 1.24, *t* = 5.71, *p* < 0.001), whereas females visited flowers with alert call broadcasts more quickly than silent ones (estimate = −0.77, *t* = −2.50, *p* = 0.019). In the corresponding white noise control, neither sex showed a latency difference between playback and silent flowers (males: estimate = −0.04, *t* = −0.13, *p* = 0.895; females: estimate = −0.06, *t* = −0.19, *p* = 0.853). When benign social calls were broadcast, males showed no latency difference between playback and silent flowers (estimate = −0.26, *t* = −0.75, *p* = 0.459), whereas females showed a weak tendency to reach playback flowers more quickly than silent flowers (estimate = −0.61, *t* = −2.04, *p* = 0.052). In the corresponding white noise control, females again showed no latency difference (estimate = −0.38, *t* = −1.10, *p* = 0.282), whereas males reached playback flowers more quickly than silent flowers (estimate = −0.62, *t* = −3.68, *p* = 0.001). Repetition did not significantly affect latency in any model (all *ps* ≥ 0.192).

Only two males—the highest ranking ones in our study—approached the flowers where alert calls were broadcast. The focal males exhibited a linear dominance hierarchy, with an alpha male at the top and a beta male below (Table S3). The alpha male visited the flower with the alert call broadcast more than twice as often as the beta male but preferred the silent flower (Figure 2A). The other six males completely avoided the flower with the alert call broadcast, visiting only the silent flower. Additionally, the alpha male had a significantly shorter latency to his first flower visit than the beta male (Figure S2A). Although females in our study also displayed a linear dominance hierarchy (Table S4), unlike the males, we did not observe any differences in their response strength across ranks.

Linear regression analyses showed that male dominance significantly predicted male response strength during alert call playbacks, explaining 78.2% of the variance (*F*_1,6_ = 21.50, *p* = 0.004, *R* ^2^ = 0.782, *n* = 8; Figure 3). Higher DSs were associated with stronger responses, that is, more flower visits. By contrast, during benign call playbacks, male dominance was unrelated to response strength, and the model explained virtually no variance (*F*_1,6_ = 0.008, *p* = 0.932, *R* ^2^ = 0.001, *n* = 8; Figure 3).

**FIGURE 3:**
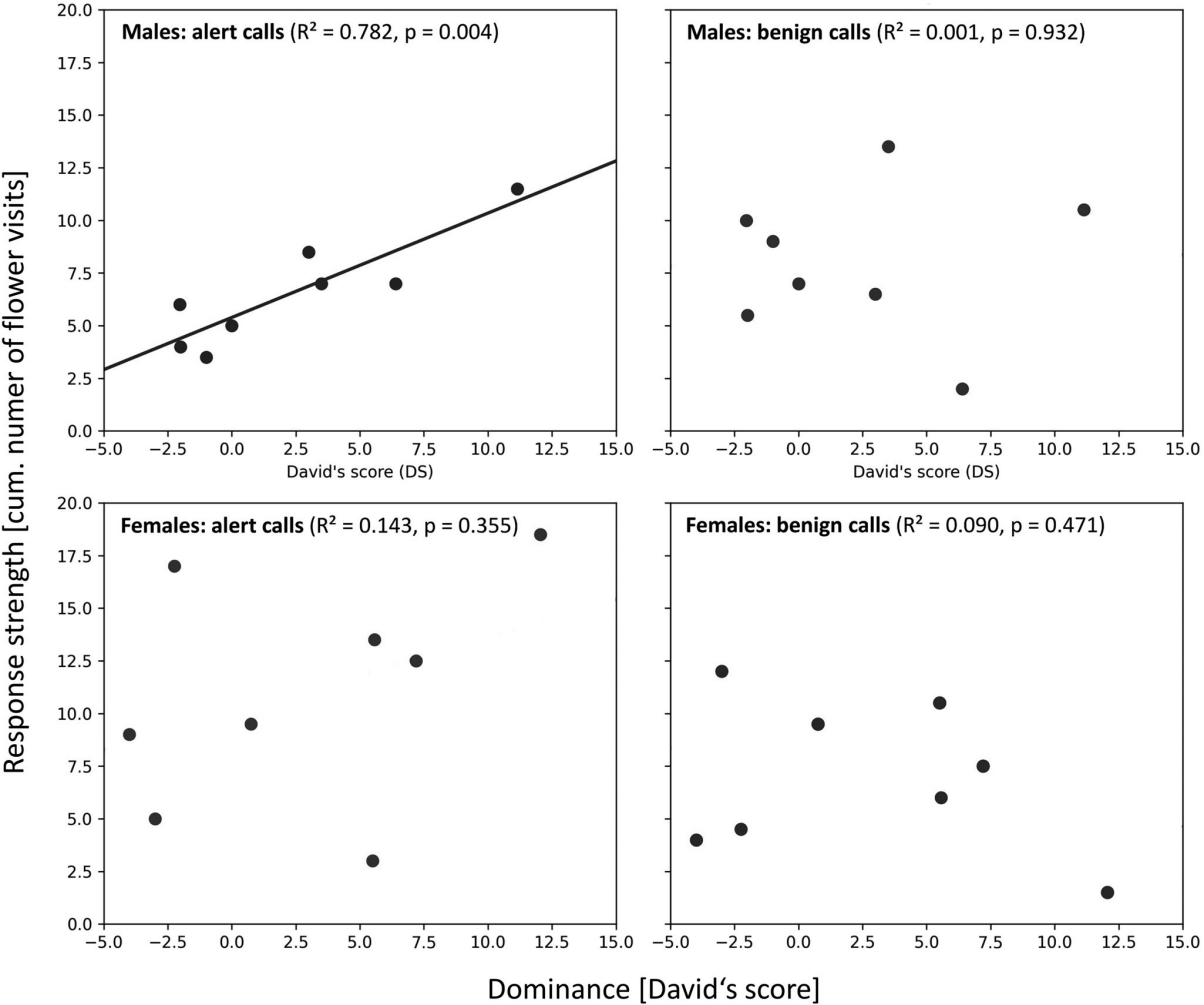
Relationship between dominance and response strength for males and females, shown separately for playbacks of alert calls and benign social calls. Each point represents an individual. The line indicates the only significant linear regression fit.

In contrast to males, female dominance was not significantly associated with female response strength during either playback type. Although the effect was weakly positive for alert call playbacks and weakly negative for benign call playbacks, both models explained little variance, and neither relationship reached significance (alert calls: *F*_1,6_ = 1.00, *p* = 0.355, *R* ^2^ = 0.143, *n* = 8; benign calls: *F*_1,6_ = 0.593, *p* = 0.471, *R* ^2^ = 0.090, *n* = 8; Figure 3).

Neither male nor female dominance were not significantly associated with latency during either playback type (males: alert calls: *F*_1,6_ = 0.361, *p* = 0.570, *R* ^2^ = 0.057; benign calls: *F*_1,6_ = 0.661, *p* = 0.447, *R* ^2^ = 0.099; females: alert calls: *F*_1,6_ = 0.485, *p* = 0.512, *R* ^2^ = 0.075; benign calls: *F*_1,6_ = 0.728, *p* = 0.426, *R* ^2^ = 0.108).

## 4 Discussion

Our findings indicate that social vocalizations influence foraging choices in *G. mutica*, and this effect varies notably between sexes. As predicted, bats altered their flower selections when exposed to alert call playbacks, but males and females responded differently: Males avoided whereas females preferred flowers associated with alert calls. As alert calls in this species are typically produced in contexts that precede aggressive encounters, male avoidance aligns with the idea that these calls signal a higher likelihood of social conflict at a feeding site [35, 38]. Conversely, females' attraction to these signals suggests that flowers paired with social calls are not valued equally by all receivers, indicating that males and females differ in how they evaluate and respond to them. For instance, females may prefer to forage in company because this could facilitate resource location or reduce predation risk, even though the presence of conspecifics may result in aggressive interactions. This discovery is important because it demonstrates that the same social signal can trigger different behavioral responses depending on the receiver's sex and, likely, the fitness consequences of responding.

Only the highest ranking males approached flowers while alert calls were being broadcast, whereas all others avoided them. Moreover, male dominance rank predicted the strength of their responses during alert call playbacks. However, because playback stimuli were compiled from unfamiliar conspecifics, we could not quantify dominance relationships between callers and receivers. Our results therefore support an association between receiver dominance and response strength but do not reveal whether responses depend on rank asymmetry between caller and receiver. Nevertheless, our results suggest that responses to conflict‐related signals may depend on competitive ability, as dominance rank likely reflects an individual's capacity to tolerate or win aggressive interactions: Higher ranking individuals are less deterred by signals indicating contested resources. This aligns with broader theoretical and empirical research showing that dominance relationships and socially mediated interference can influence access to feeding opportunities and change how individuals exploit resources associated with potential competition. Among social foragers, subordinates often face higher costs when dominants are nearby, and these differences can affect both movement and patch choice [48]. Similar patterns have been documented across taxa, from primates, where high‐ranking individuals gain priority access to food resources, to social carnivores, where access to carcasses is influenced by dominance and competitive ability, with subordinates experiencing fewer feeding opportunities [49, 50, 51]. More broadly, social context can alter decision‐making strategies by shifting the perceived balance between risk and reward, especially during competition [52, 53]. In our focal species, alert calls were associated with situations of potential social conflict, but only males with sufficient status or competitive ability appeared willing to see these contested flowers as worthwhile opportunities rather than sites to avoid.

Work on other bats shows that social calls produced during foraging can serve functions ranging from recruitment and coordination to resource defense. In *Phyllostomus hastatus*, low‐frequency screech calls attracted bats at both roost and feeding sites and coordinated females from the same roosting group during the exploitation of concentrated resources, potentially facilitating collective resource defense [54]. More recently, food‐associated calls produced by feeding *Thyroptera tricolor* increased feeding‐related behavior and attracted flying conspecifics, providing another example of vocal recruitment to food resources [55]. By contrast, social calls can also deter competitors: In *Pipistrellus pipistrellus*, calling increased as insect availability declined, and playback reduced the activity of bats of the same phonic type [9]. Further evidence suggests graded resource defense, with complex social calls used at longer range and high‐frequency calls during close chases and competitor displacement [56]. Male *Eptesicus fuscus* likewise used individually distinctive calls to claim prey, thus improving the caller's capture success [57]. Similarly, in female *Vespertilio sinensis*, social calling increased when food was limited, call rate covaried with dominance and weight gain, and playback reduced visits and consumption at a food dish, consistent with honest signaling of competitive ability and food defense [58]. Playbacks of foraging‐associated social calls also reduced foraging effort and prey consumption in female *Myotis macrodactylus*, supporting a deterrent function during competition [59]. Nevertheless, aggressive calls do not always repel receivers: Male *Murina leucogaster* preferred food trays paired with antagonistic calls over trays paired with echolocation calls or white noise, suggesting that such calls can signal the presence or value of a resource [60]. Our findings extend previous work by showing that responses to conflict‐related calls depend on receiver phenotype: Alert calls attracted females but deterred most males, whose responses also varied with dominance rank. Thus, competitive ability can shape not only signal production but also signal evaluation, whereas attraction to antagonistic calls can be sex‐specific rather than general. Moreover, by pairing calls with one of two otherwise equivalent flowers and testing both sexes with alert and benign calls, we measured the relative value assigned to social information rather than only overall attraction to or suppression at a single feeding site.

In contrast to our expectations, alert calls showed no structural differences between males and females, indicating that the signal itself does not encode sex‐specific information. This is a key finding because it demonstrates that the behavioral divergence we observed does not arise from signal dimorphism but from sex‐specific interpretation of the same signal. Similarly, the trills used by several pipistrelle species in Europe are employed to deter conspecifics at foraging sites, but males also use them during song flight behavior in the mating season to attract females [9, 10, 11]. Therefore, opposite functional effects in bat social vocalizations do not necessarily require distinct signal structures. Recent work on *G. mutica* further suggests that social vocalizations may convey information beyond immediate context, as alert call production is associated with proactive, exploratory, and bold behavioral types in males [38], potentially influencing how receivers evaluate the costs and benefits of interacting at socially active sites. More generally, this supports a receiver‐dependent perspective on communication, in which the meaning and behavioral value of a signal depend on the interaction among the signal, context, and the receiver's state, rather than on the signal's design alone. Experimental work in other taxa similarly shows that males and females can derive different information from the same signal, even in the absence of sex‐specific structural differences. Different receivers may interpret distinct components of a complex display, resulting in divergent behavioral responses [61, 62]. More generally, variation in perception and decision‐making rules among receivers can shape how a given signal is interpreted and used [63].

The comparison between alert and benign calls further clarifies this interpretation. In line with our expectations, responses to benign calls differed from those to alert calls. Males no longer avoided the playback flower, whereas females continued to prefer it. This indicates that male avoidance is not a general reaction to conspecific acoustic activity but is specifically linked to signals associated with antagonistic or conflict‐prone situations. Females, in contrast, responded positively not only to alert calls but also to benign calls, showing a broader attraction to feeding sites with social activity.

Importantly, these results suggest that sex‐specific responses arise from a shared, context‐dependent signaling system, in which alert and benign calls convey different information about social contexts, but males and females vary in how they interpret and react to this information. Males appear to perceive alert calls as signs of competition for flowers and potential disturbance, whereas females appear to respond positively to vocalizations from group members, even in potentially aggressive situations.

A simple explanation is that the two sexes prioritize information differently regarding the risks of social competition. In many social animals, shared or incidental social information can help locate resource patches and reduce search effort, but this benefit is balanced by increased competition, interference, or harassment at popular sites [48, 53]. In nectar‐feeding bats, including *Glossophaga*, individuals can use social information to locate profitable flowers, for example, via local enhancement or by eavesdropping on the activity of conspecifics, thereby reducing search effort [26, 27]. These forms of information rely primarily on cues generated by others' behavior, whereas, in our study, bats responded to intentional social signals; nevertheless, both can inform decisions about where to forage. At the same time, these bats may defend floral resources and actively exclude competitors, indicating that socially active flowers can simultaneously represent foraging opportunities and sites of competition [31].

In insectivorous bats, acoustic information related to foraging can provide valuable public information: Individuals may eavesdrop on the echolocation activity of others, especially on feeding buzzes, which function as inadvertent cues of prey detection, to find profitable foraging opportunities [64, 65, 66, 67]. However, socially active sites can also entail high costs, as increasing local bat density may raise interference, competition, and sensory overload [68, 69, 70]. Although the vocalizations we studied are social signals rather than incidental foraging cues, such as feeding buzzes, responses to the latter show that acoustic activity at foraging sites can indicate both opportunities and costs, suggesting that receivers can use acoustic information more generally to assess the profitability of socially active patches. In our system, females responded positively to this social information, whereas males mostly avoided the potential competitive costs signaled by conspecific vocalizations. This suggests that males and females assess these trade‐offs differently: For males, particularly non‐dominant individuals, signals of conflict may reduce the perceived value of feeding sites, whereas females may continue to exploit the informational benefits of conspecific activity even when those sites are contested.

More broadly, the lack of structural differences in the signal, combined with clear sex‐specific responses and their context dependence, suggests that sex‐specific behavioral differences can develop without sex‐specific signal design. The key distinction, therefore, is not in the acoustic structure of the signal but in how different receivers interpret its function. This insight is important for understanding a significant aspect of social communication: Selection need not produce sex‐specific call variants to cause sex‐specific behavioral outcomes. Instead, divergence may occur at the level of receiver perception, decision‐making rules, or social payoff structures. In our case, this divergence is consistent with differences in how males and females respond to social competition, as well as in their expected benefits from engaging with conspecifics at feeding sites. Future research should therefore go beyond signal structure alone and explore the proximate and ultimate causes of receiver‐dependent responses, including the roles of dominance, mating strategies, prior social experiences, and reproductive states.

## Author Contributions

Conceptualization: Mirjam Knörnschild. Data acquisition: Mirjam Knörnschild. Formal analysis and investigation: Mirjam Knörnschild and Danilo Russo. Writing – original draft preparation: Danilo Russo. Writing – review and editing: Mirjam Knörnschild and Danilo Russo. Funding acquisition: Mirjam Knörnschild and Danilo Russo.

## Conflicts of Interest

The authors declare no conflicts of interest.

## Supporting information




**Supplementary Material**: nyas70427‐sup‐0001‐SuppMat.docx

**Supplementary Material**: nyas70427‐sup‐0002‐DataS1.xlsx

**Supplementary Material**: nyas70427‐sup‐0003‐DataS2.xlsx

**Supplementary Material**: nyas70427‐sup‐0004‐VideoS1.mp4

**Supplementary Material**: nyas70427‐sup‐0005‐VideoS2.mp4

**Supplementary Material**: nyas70427‐sup‐0006‐VideoS3.mp4

## Data Availability

The data that support the findings of this study are available from the corresponding author upon request.
