# Supplementary material for: Sex‐Specific Responses to Social Calls Shape Foraging Decisions in a Nectar‐Feeding Bat
Source: Ann N Y Acad Sci. 2026 Oct 21;1564(1):e70427. doi: 10.1111/nyas.70427 (PMC13686553; doi:10.1111/nyas.70427)
Supplement: Supplementary file 1 [file NYAS-1564-0-s002.docx]

**SUPPLEMENTARY MATERIAL**

**Sex-specific responses to social calls shape foraging decisions in a nectar-feeding bat**

Mirjam Knörnschild, Danilo Russo

**Fig. S1:** Experimental set-up for experiment 1 and 2, consisting of four trials each.

**Fig. S2:** Average latency to the first flower visit of focal bats

**Table S1:** Repeatability analyses for preferences of *G. mutica* in playback experiments

**Table S2:** Matrix of dominance interactions of *G. mutica* dyads

**Table S3:** Matrix of dominance interactions of *G. mutica* female dyads

**Table S4:** David’s scores for focal male and female *G. mutica*

**Table S5:** Mean acoustic parameters of alert call types from male and female *G. mutica*

**Data S1**: Acoustic parameters of alert call types from male and female *G. mutica*

**Data S2**: Playback response data (flower visits and latency) from male and female *G. mutica*

**Video S1**: Dyadic interaction between male 1 and male 3 (chasing).

**Video S2**: Dyadic interaction between male 1 and male 2 (displacement).

**Video S3**: Dyadic interaction between male 3 and male 4 (chasing).

**Fig. S1**: Experimental set-up for experiment 1 (alert calls) and 2 (benign calls), consisting of four trials each. During the first trial, the speaker was located over one artificial flower, broadcasting alert calls (or benign calls), and the dummy speaker was placed over the second artificial flower, functioning as a silent control. During the second trial, the artificial flowers and speakers were relocated to opposite corners of the flight cage; the speaker broadcast white noise, and the dummy speaker functioned as a silent control. Afterwards, both trials were repeated with a spatially reversed stimulus presentation to control for a potential side preference of our focal bats.

**Fig. S2**: Average latency to the first flower visit of focal bats during two different playback experiments. Males visited silent flowers faster than flowers at which alert calls were broadcast (**A**), whereas females tended to visit flowers with alert call broadcasts faster than silent ones (**B**). Playback of benign calls did not influence males’ (**C**) and females’ (**D**) foraging behaviour.

**Repeatability analysis**

To assess individual variation across repeated measurements, we calculated per-repetition preference scores for each bat separately for flower visits and latency. For flower visits, preference was defined as the log-ratio of visits to the playback flower relative to the silent flower; for latency, preference was defined as the log-ratio of latency to the silent flower relative to latency to the playback flower, such that positive values indicated faster approach to the playback flower. We then quantified consistency between repetition 1 and repetition 2 within each sex × call type × playback condition (stimulus or noise control) using Spearman rank correlations and intraclass correlation coefficients (ICC). ICCs evaluate how similar repeated measurements from the same individual are, relative to the variation among individuals (an ICC close to 1 means that bats show very similar preference scores in both repetitions).

Preference scores were generally repeatable across the two repetitions, indicating consistent individual responses. Repeatability was especially high in the alert call experiment: both females and males showed strong consistency across repetitions for flower visit preference and latency preference in the social-call condition, and repeatability was likewise high in the corresponding white noise controls. Repeatability in the benign call experiment was more variable, with the weakest consistency observed in females. Overall, these analyses show that the pronounced sex-specific responses to alert calls were not driven by inconsistent behaviour across repeated measurements.

**Table S1**: Repeatability analyses for preferences of *G. mutica* in playback experiments

**Table S2**: Matrix of dominance interactions of *G. mutica* male dyads.

**Table S3**: Matrix of dominance interactions of *G. mutica* female dyads.

**Table S4**: David’s scores for focal male and female *G. mutica*

| **Bat** | **DS** |
| --- | --- |
| **male 1** | 11.14 |
| **male 2** | 6.39 |
| **male 3** | 3.5 |
| **male 4** | 3 |
| **male 5** | -2.04 |
| **male 6** | -1 |
| **male 7** | 0 |
| **male 8** | -2 |
| **female 1** | 12.05 |
| **female 2** | 7.2 |
| **female 3** | 5.57 |
| **female 4** | 5.5 |
| **female 5** | 0.75 |
| **female 6** | -2.25 |
| **female 7** | -4 |
| **female 8** | -3 |

David’s scores were calculated from the matrix of observed winner–loser interactions (Tables S2 and S3) using the *DS()* function of the R package *EloRating*.

**Table S5**: Acoustic parameters of alert call types from male and female *G. mutica*
